# Supplementary material for: The Prospective COVID-19 Post-Immunization Serological Cohort in Munich (KoCo-Impf): Risk Factors and Determinants of Immune Response in Healthcare Workers
Source: Viruses. 2023 Jul 18;15(7):1574. doi: 10.3390/v15071574 (PMC10383736; doi:10.3390/v15071574)
Supplement: Supplementary file 1 [file viruses-15-01574-s001.zip › viruses-2478429-supplementary.pdf]

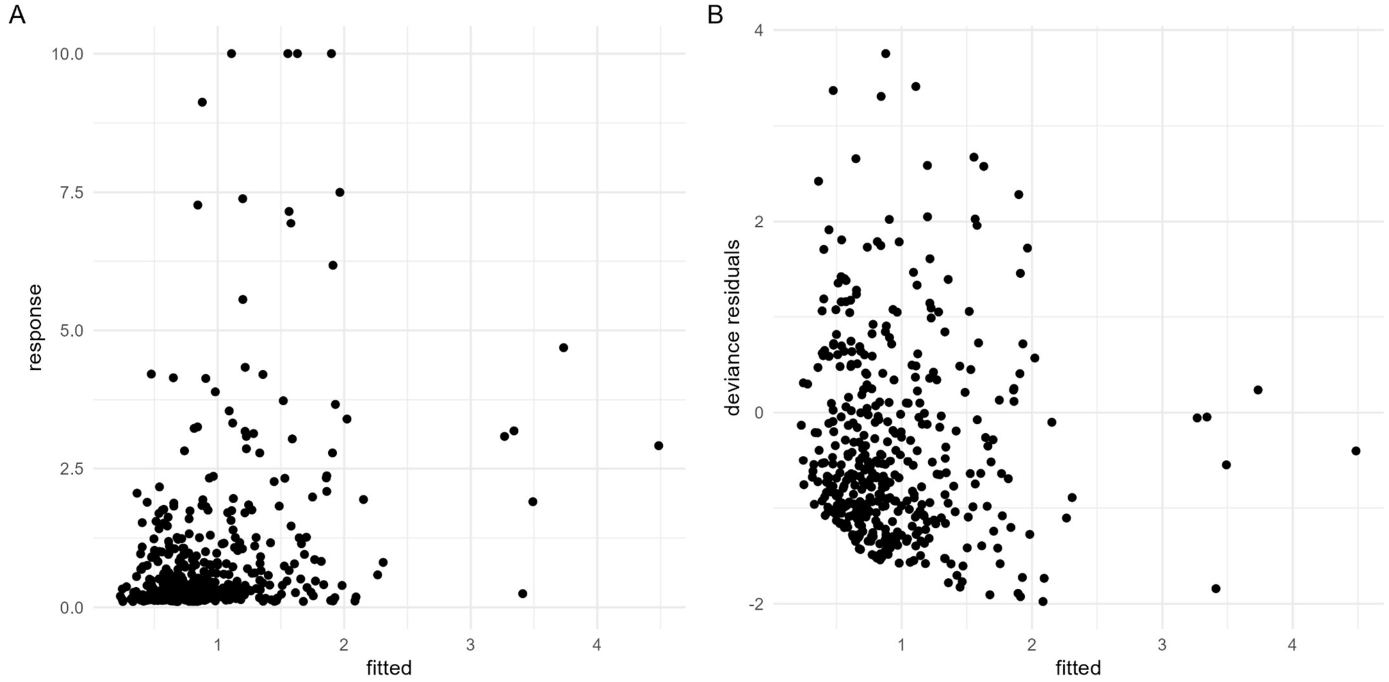

Figure S1. Model check for Anti-N quantity, based on positive anti-N serology. Results are based on one imputed dataset as an example and are derived from a generalized linear model (GLM) with gamma distribution. Differences between the dataset are present but not relevant (see CI) and are due to the fact that for 68% (286/424) of the observations the time since infection variable was missing and therefore imputed.

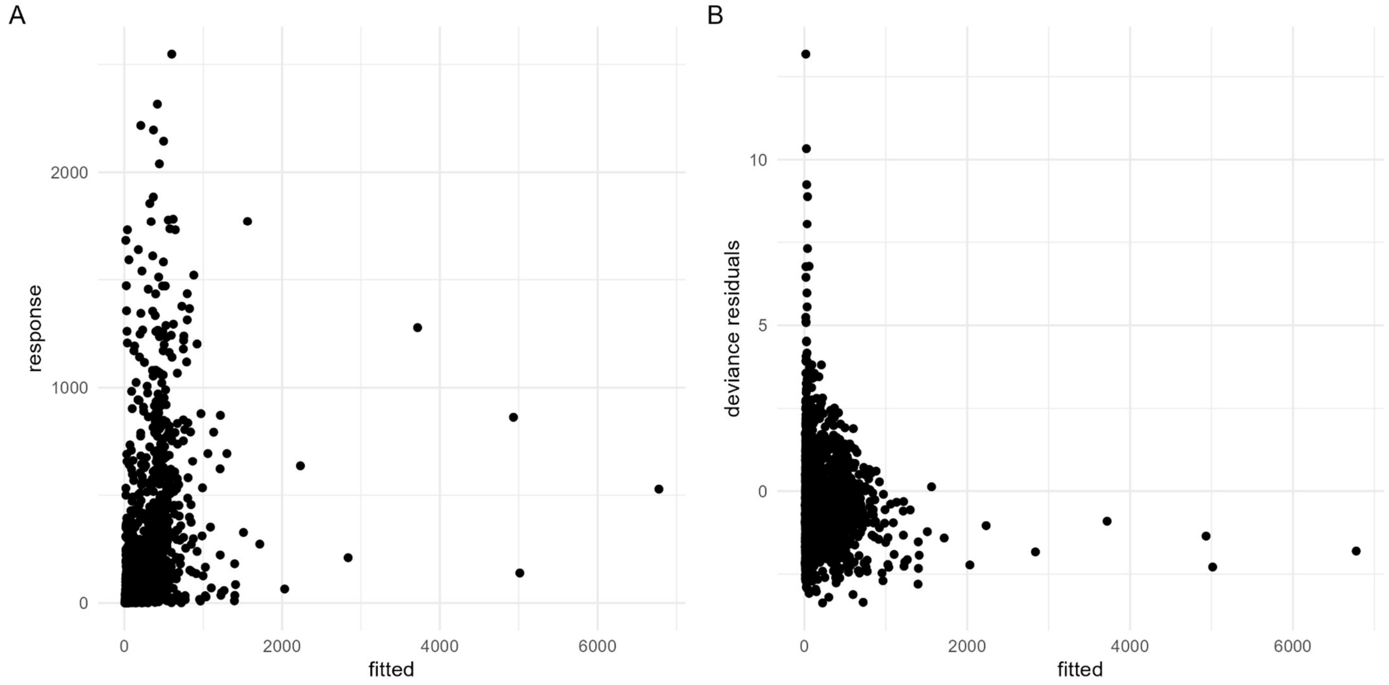

Figure S2. Model check for Anti-S quantity, based on positive anti-S serology. Results are based on one imputed dataset as an example and are derived from a generalized linear model (GLM) with gamma distribution.

Table S1. Values to the risk factor analysis for SARS-CoV-2 infection, based on positive anti-N serology. Results are based on a logistic regression model and are given as the logarithms of the ORs with their 95% CI.

**Covariate**	**Category**	**Logistic Regression Model Qualitative Analysis of Binary Anti-N**		
		**Log (OR)**	**95% CI**	**P-value**
Institutional subgroup	General population	1		
	Barmherzige Brüder	3.84	(3.09;4.59)	**<0.001*** **
	Eichenau	3.84	(2.56;5.12)	**<0.001*** **
	Friedenheimer Brücke	1.76	(-0.38;3.92)	0.11
	Medical center of LMU	2.15	(1.44;2.86)	**<0.001*** **
	MK, Bogenhausen	2.30	(1.50;3.10)	**<0.001*** **
	MK, Harlaching	2.27	(1.37;3.17)	**<0.001*** **
	MK, Neuperlach	1.65	(0.48;2.81)	**0.005** **
	MK, Schwabing	1.77	(0.89;2.65)	**<0.001*** **
	MK, Thalkirchner Straße	2.06	(0.77;3.34)	**0.002** **
	MS, Heilig Geist	3.39	(2.38;4.40)	**<0.001*** **
	MS, Rümannstraße	1.85	(-0.004;3.71)	**0.05***
	Obersendling	2.74	(1.26;4.21)	**<0.001*** **
	Seefeld	2.34	(1.11;3.57)	**<0.001*** **
	Tropical Institute	1.35	(-0.30;3.01)	0.11
	Vaccination center Riem	2.44	(1.69;3.18)	**<0.001*** **
Age		0.002	(-0.006;0.01)	0.59
Sex	Female	1		
	Male	0.04	(-0.15;0.28)	0.70
Contact with patients	No	1		
	Yes	0.13	(-0.15;0.41)	0.36
Contact with positives	No or unwittingly	1		
	Yes	0.79	(0.55;1.04)	**<0.001*** **
Smoking status	Never smoker	1		
	Current smoker	-0.67	(-1.01;-0.33)	**<0.001*** **
	Past smoker	-0.18	(-0.52;0.14)	0.26
Vaccination scheme	No vacc.	1		
	One vacc.	-0.50	(-1.14;0.14)	0.12
	Two vacc.	-3.63	(-4.33;-2.93)	**<0.001*** **
	Three vacc.	-3.90	(-4.80;-3.01)	**<0.001*** **
Household size	One person	1		
	2 people	-0.19	(-0.47;0.08)	0.17
	3 people	-0.03	(-0.37;0.30)	0.84
	4 people	-0.01	(-0.35;0.33)	0.93
	5 people or more	-0.07	(-0.57;0.42)	0.76
Intake of immunosuppressive drugs	No	1		
	Yes	-0.32	(-1.01;0.37)	0.36
Cumulative cases		0.92	(-0.17;2.02)	0.10

OR: Odds Ratio

Table S2. Values to the association between quantitative anti-N serology and determinants of antibody response. Results are based on a GLM with gamma distribution and are given as coefficients of the model with their 95% CI.

**Covariate**	**Category**	**Generalized Linear Models (GLM) Quantitative Analysis of Positive Anti-N**		
		**Coefficient**	**95% CI**	**P-value**
Age		0.01	(0.003;0.02)	**0.01***

Sex	Female	1		
	Male	0.22	(-0.08;0.53)	0.15
Smoking status	Never smoker	1		
	Current smoker	-0.43	(-0.86;-0.005)	**0.05***
	Past smoker	0.10	(-0.32;0.52)	0.63
Vaccination scheme	No vacc.	1		
	One vacc.	-0.41	(-1.03;0.22)	0.20
	Two vacc.	-0.76	(-1.43;-0.09)	**0.03***
	Three vacc.	-1.00	(-1.95;-0.05)	**0.04***
Intake of immunosuppressive drugs	No	1		
	Yes	0.12	(-0.80;1.03)	0.79
Time since infection	Less than three months ago	1		
	Three to less than six months ago	0.65	(-2.28;3.58)	0.58
	Six to twelve months ago	0.31	(-0.56;1.19)	0.46
	More than twelve months ago	-0.04	(-1.00;0.91)	0.92
BTI	No	1		
	Yes	-0.04	(-0.75;0.65)	0.89
Cumulative cases		0.65	(-0.62;1.93)	0.30

Table S3. Values to the association between quantitative anti-S serology and determinants of antibody response. Results are based on a GLM with gamma distribution and are given as coefficients of the model with their 95% CI.

**Covariate**	**Category**	**Generalized Linear Models (GLM) Quantitative Analysis of Positive Anti-S**		
		**coefficient**	**95% CI**	**P-value**
Age		-0.01	(-0.01;-0.007)	**<0.001*****
Sex	Female	1		
	Male	-0.04	(-0.17;0.08)	0.49
Smoking status	Never smoker	1		
	Current smoker	-0.21	(-0.36;-0.06)	**0.004****
	Past smoker	0.003	(-0.17;0.18)	0.97
Intake of immunosuppressive drugs	No	1		
	Yes	0.12	(-0.21;0.45)	0.47
Time since infection	Less than three months ago	1		
	Three to less than six months ago	-0.31	(-1.60;0.98)	0.63
	Six to twelve months ago	0.42	(-0.50;1.36)	0.36
	More than twelve months ago	0.26	(-0.70;1.24)	0.58
	No infection	-0.80	(-1.86;0.24)	0.13
BTI	No	1		
	Yes	1.40	(0.80;2.00)	**<0.001*****
Immunity	Infection yes, not vaccinated	1		
	Infection yes+ one vaccination	2.76	(1.84;3.69)	**<0.001*****
	Infection yes+ two vaccinations	3.93	(3.04;4.82)	**<0.001*****
	Infection yes+ three vaccinations	4.40	(3.02;5.78)	**<0.001*****
	Infection no+ one vaccination	1.49	(0.47;2.50)	**<0.001*****
	Infection no+ two vaccinations	3.15	(2.13;4.17)	**<0.001*****
	Infection no+ three vaccinations	6.15	(5.09;7.21)	**<0.001*****
Time since second vaccination		-0.006	(-0.007;-0.005)	**<0.001*****
Cumulative cases		0.93	(0.52;1.34)	**<0.001*****

Table S4. Sensitivity analysis of the risk factor analysis for SARS-CoV-2 infection, based on positive anti-N serology, excluding the variable "institutional subgroup". Results are based on a logistic regression model and are given as the logarithms of the ORs with their 95% CI.

Covariate	Category	Logistic Regression Model Qualitative Analysis of Binary Anti-N		
		**Log (OR)**	**95% CI**	**P-value**
Age		0.005	(-0.003;0.01)	0.22
Sex	Female	1		
	Male	-0.01	(-0.25;0.20)	0.91
Contact with patients	No	1		
	Yes	0.52	(0.14;0.69)	**<0.001*******
Contact with positives	No or unwittingly	1		
	Yes	0.91	(0.70;1.17)	**<0.001*******
Smoking status	Never smoker	1		
	Current smoker	-0.55	(-0.87;-0.23)	**<0.001*******
	Past smoker	-0.18	(-0.49;0.13)	0.26
Vaccination scheme	No vacc.	1		
	One vacc.	0.80	(0.47;1.34)	**<0.001*******
	Two vacc.	-1.79	(-2.10;-1.20)	**<0.001*******
	Three vacc.	-2.13	(-2.71;-1.29)	**<0.001*******
Household size	One person	1		
	2 people	-0.17	(-0.44;0.09)	0.20
	3 people	0.01	(-0.32;0.32)	0.93
	4 people	-0.03	(-0.36;0.29)	0.86
	5 people or more	-0.09	(-0.53;0.44)	0.72
Intake of immunosuppressive drugs	No	1		
	Yes	-0.18	(-0.89;0.42)	0.58
Cumulative cases		1.20	(0.41;1.89)	**0.002****
